# Fhit, a tumor suppressor protein, induces autophagy via 14-3-3τ in non-small cell lung cancer cells

**DOI:** 10.18632/oncotarget.16652

**Published:** 2017-03-29

**Authors:** Tae-Gul Lee, Eun-Hui Jeong, Seo Yun Kim, Hye-Ryoun Kim, Hyunggee Kim, Cheol-Hyeon Kim

**Affiliations:** ^1^ Division of Pulmonology, Department of Internal Medicine, Korea Cancer Center Hospital, Seoul, Korea; ^2^ School of Life Sciences and Biotechnology, Korea University, Seoul, Korea

**Keywords:** Fhit, autophagy, 14-3-3τ, non-small cell lung cancer, apoptosis

## Abstract

Inactivation of the fragile histidine triad (*Fhit*) gene has been reported in the majority of human cancers, particularly in lung cancer. The role of *Fhit* as a tumor suppressor gene has been well documented, and restoration of *Fhit* expression suppresses tumorigenicity in tumor cell lines and in mouse models by inducing apoptosis and inhibiting proliferation of tumor cells. Autophagy is a catabolic pathway, whereby cytoplasmic proteins and organelles are sequestered in vacuoles and delivered to lysosomes for degradation and recycling. Although autophagy is necessary for cell survival under stress conditions, recent studies have shown that autophagy can also promote cell death. Due to the fact that both autophagy induction and Fhit expression are commonly associated with nutrient starvation, we hypothesized that Fhit expression may be related to autophagy induction. In the present study, we assessed whether Fhit overexpression by gene transfer induces autophagy in Fhit-deficient non-small cell lung cancer (NSCLC) cells. The results of our study indicate that Fhit protein induces autophagy in NSCLC cells, and that this autophagy prevents apoptotic cell death *in vivo* and *in vitro* in a 14-3-3τ protein-dependent manner. To the best of our knowledge, this is the first report to describe Fhit-induced autophagy. Suppressing autophagy might be a promising therapeutic option to enhance the efficacy of *Fhit* gene therapy in NSCLC.

## INTRODUCTION

The fragile histidine triad (*Fhit*) gene is located on chromosome 3p14.2 and encompasses the FRA3B fragile site, one of the most fragile sites in the human genome [1]. Inactivation of *Fhit* gene by deletion, decreased expression, or promoter methylation has been reported in the majority of human cancers, particularly in lung cancer [2–5]. The role of *Fhit* as a tumor suppressor gene has been well documented. Restoration of *Fhit* expression suppresses tumorigenicity in tumor cell lines and in mouse models by inducing apoptosis and inhibiting proliferation of tumor cells [5–10], suggesting that *Fhit* gene therapy could constitute a novel therapeutic approach for cancer treatment [11].

Autophagy is a catabolic pathway, whereby cytoplasmic proteins and organelles are sequestered in vacuoles and delivered to lysosomes for degradation and recycling. Environmental stressors, such as nutrient starvation, pathogen infection, high temperature, and low oxygen, can induce autophagy [12–15]. In the early stages of autophagy, portions of the cytoplasm, as well as intracellular organelles, are sequestered in double-membrane-bound structures known as autophagosomes. These autophagosomes then fuse with lysosomes to form autolysosomes, and the sequestered contents are degraded by lysosomal hydrolases and their components are recycled [12–15]. Although autophagy is necessary for cell survival under stress conditions, recent studies have shown that autophagy can also promote cell death [16–18]. It is unclear which autophagy contexts promote cell death versus cell survival.

Previous studies have shown increased Fhit protein levels after serum starvation of lung and breast cancer cells as seen by Western blotting and immunocytochemical assays [8, 19]. Both autophagy induction and Fhit expression are commonly associated with nutrient starvation, so we hypothesized that Fhit expression may be related to autophagy induction. The relationship between Fhit and autophagy has not yet been investigated. In this study, we examined if Fhit expression is related to autophagy and showed that Fhit indeed induces autophagy, and that this autophagy is dependent on the 14-3-3τ protein and prevents apoptotic cell death in non-small cell lung cancer (NSCLC) cells.

## RESULTS

### Endogenous Fhit expression is associated with starvation-induced autophagy in NSCLC cells

**Figure 1:**
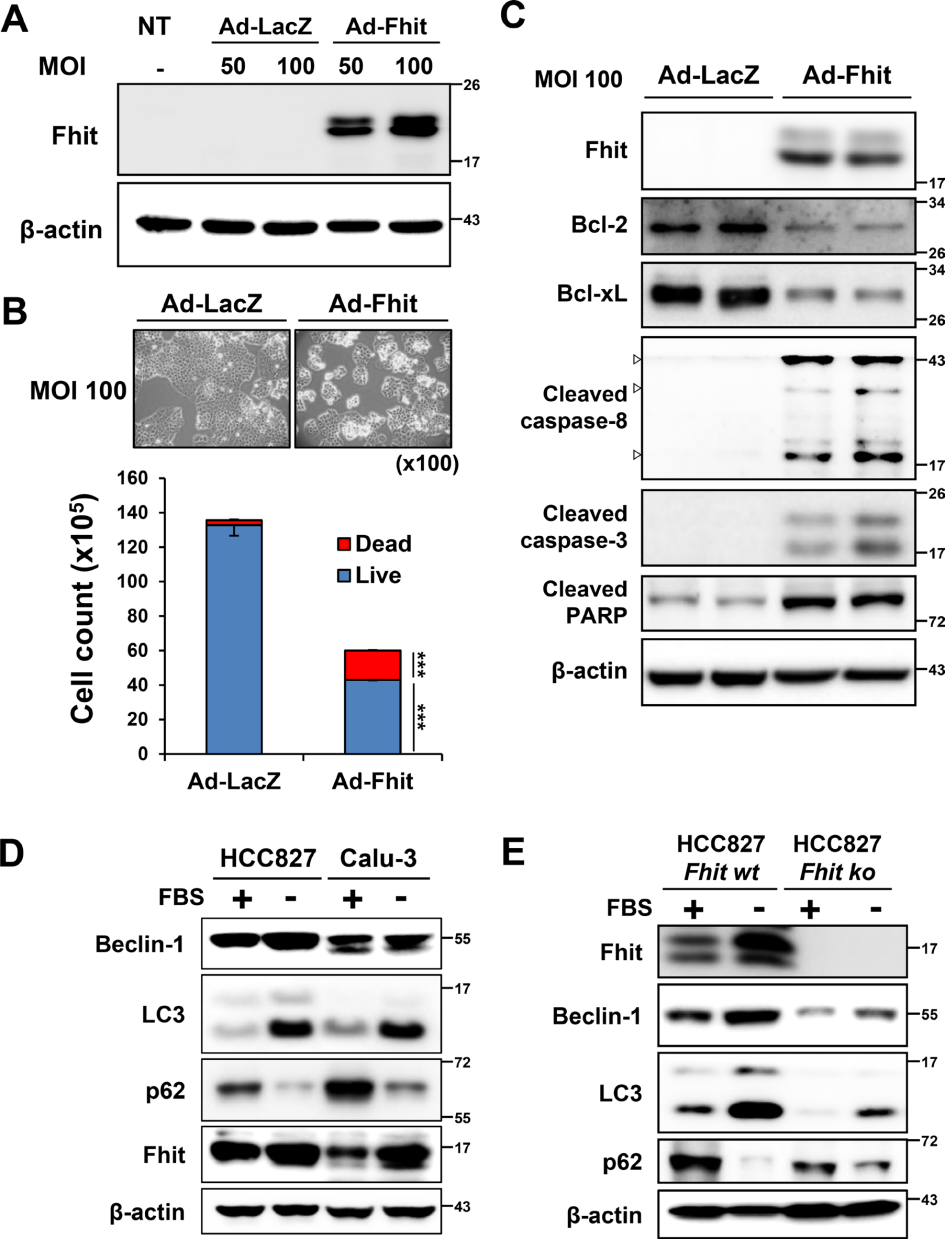
Starvation-induced autophagy and up-regulation of Fhit in NSCLC cells (**A**–**C**) Fhit overexpression activates caspase-dependent apoptotic pathway in NSCLC cells. H460 cells were transduced with Ad-Fhit (A). Two days after transduction, apoptosis was examined by trypan blue staining (B) and Western blot analysis (C). *LacZ* was used as a negative control. MOI, multiplicity of infection; NT, not treated. ***p < 0.001. (**D**) Serum starvation induces autophagy and Fhit is up-regulated during this process. HCC827 and Calu-3 cells were kept in normal culture conditions (10% FBS, +) or serum starved (−) and then cell lysates were analyzed by Western blotting with specific antibodies. (**E**) The effect of Fhit knockout on autophagy induced by serum deprivation. Endogenous Fhit was knocked out using a CRISPR/Cas9 knockout (KO) plasmid and autophagy marker proteins were analyzed by Western blotting after 24 h of serum deprivation in HCC827 cells. *wt*, wild type; *ko*, knockout.

We constructed a recombinant adenoviral-*Fhit* gene (Ad-Fhit) vector and transduced Fhit-deficient H460 lung cancer cells. Restoration of Fhit protein induced caspase-dependent apoptosis in accordance with previous reports (Figure 1A–1C). Next, we examined the effects of serum starvation on autophagy and Fhit expression in HCC827 and Calu-3 cells which express endogenous Fhit. During autophagy, cytosolic LC3-I is converted to LC3-II through lipidation, and p62 is degraded following an increase in autophagic flux. Beclin-1 has a central role in initiating autophagy [20, 21]. Serum deprivation up-regulated LC3-II and down-regulated p62, indicating autophagy induction. Interestingly, Fhit was also up-regulated during this process (Figure 1D). To examine the relationship between Fhit expression and autophagy, we compared the level of autophagy marker proteins between HCC827 cells endogenously expressing Fhit to HCC827 cells with *Fhit* stably knocked out by a CRISPR/Cas9 KO plasmid. Expression of LC3-II and degradation of p62 decreased in *Fhit*-knockout cells compared with parental cells, suggesting a critical role for Fhit in starvation-induced autophagy (Figure 1E). Expression of beclin-1 also decreased in *Fhit*-knockout cells. We also compared the level of starvation-induced autophagy between Fhit-positive (Calu-3 and HCC827) and Fhit-negative lung cancer cell lines (H358, H460, and H1299). Induction of autophagy was remarkably lower in Fhit-negative cells upon serum deprivation (Supplementary Figure 1).

### Exogenous Fhit protein expression induces autophagy in Fhit-deficient lung cancer cells

**Figure 2:**
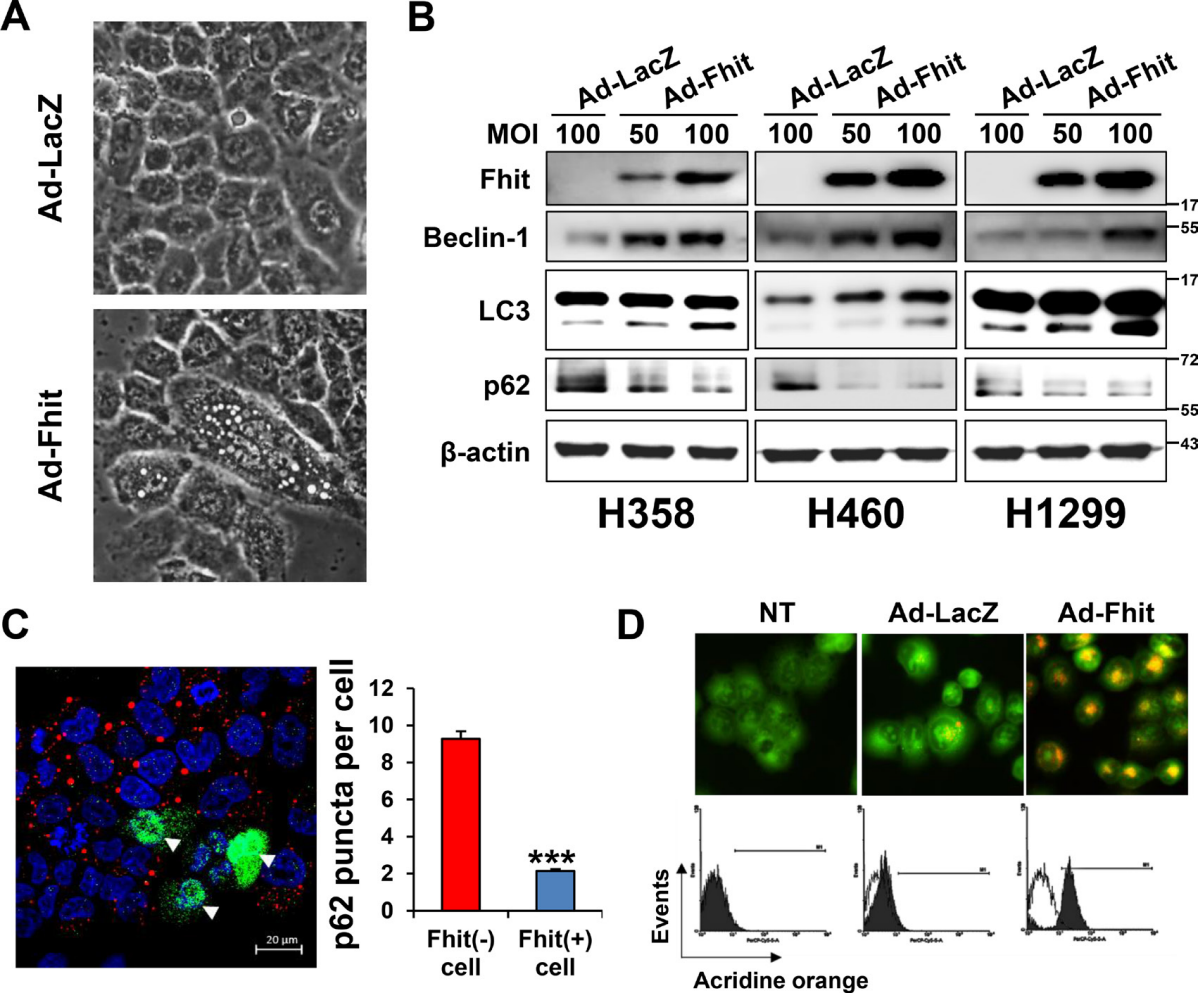
Induction of autophagy in Ad-Fhit-transduced NSCLC cells (**A**) Effect of Ad-Fhit transduction on morphology of H460 cells as shown by light microscopy (magnification ×400). (**B**) Effect of *Fhit* gene transduction on expression of autophagy marker proteins in Fhit-deficient NSCLC cells. Autophagy marker proteins were assessed by Western blot analysis 48 h after infection. Ad-LacZ-transduced cells were used as a nonspecific control for adenoviral vector-mediated gene transfer. MOI, multiplicity of infection. (**C**) Assessment of autophagy with immunofluorescence. Fhit and p62 were co-immunostained 48 h after infection with Ad-Fhit in H460 cells (left panel). Nuclei were stained with Hoechst 33342 (blue). Expression of Fhit protein is shown in green, and expression of p62 protein is shown in red. Arrowheads, cells infected with Ad-Fhit. The number of p62 puncta was determined (right panel). Results are presented as mean ± standard deviation of four independent experiments. ***p < 0.001. (**D**) Acridine orange staining 48 h after infection with Ad-Fhit or Ad-LacZ into H460 cells. Acidic vesicular organelles (AVO) were analyzed by confocal fluorescent microscopy (×400, top) and FACS analysis (bottom). NT, not treated.

Ad-Fhit was transduced into the Fhit-negative lung cancer cell lines H358, H460, and H1299 to explore the potential role of Fhit in autophagy induction. H460 cells transduced with Ad-Fhit showed morphological changes. Enlarged cells with notable vacuoles which were not detected in the control cells were visually observed by microscopy (Figure 2A). We speculated that those vacuoles might be autophagosomes. Immunoblotting revealed that Fhit expression caused a marked increase in beclin-1 and LC3-II levels and increased degradation of p62 protein compared with Ad-LacZ-infected control cells (Figure 2B). In addition, it was confirmed that p62 degradation specifically occurred in Fhit-expressing cells by an immunofluorescence assay (Figure 2C). Formation of acidic vesicular organelles (AVO), a morphological characteristic of autophagy [20, 21], was also quantified by acridine orange staining. We observed significantly increased AVO in Ad-Fhit-infected cells (Figure 2D). These findings show that Fhit protein plays a crucial role in autophagy induction in NSCLC cells.

### Fhit overexpression up-regulates a 14-3-3 protein in NSCLC cells

**Figure 3:**
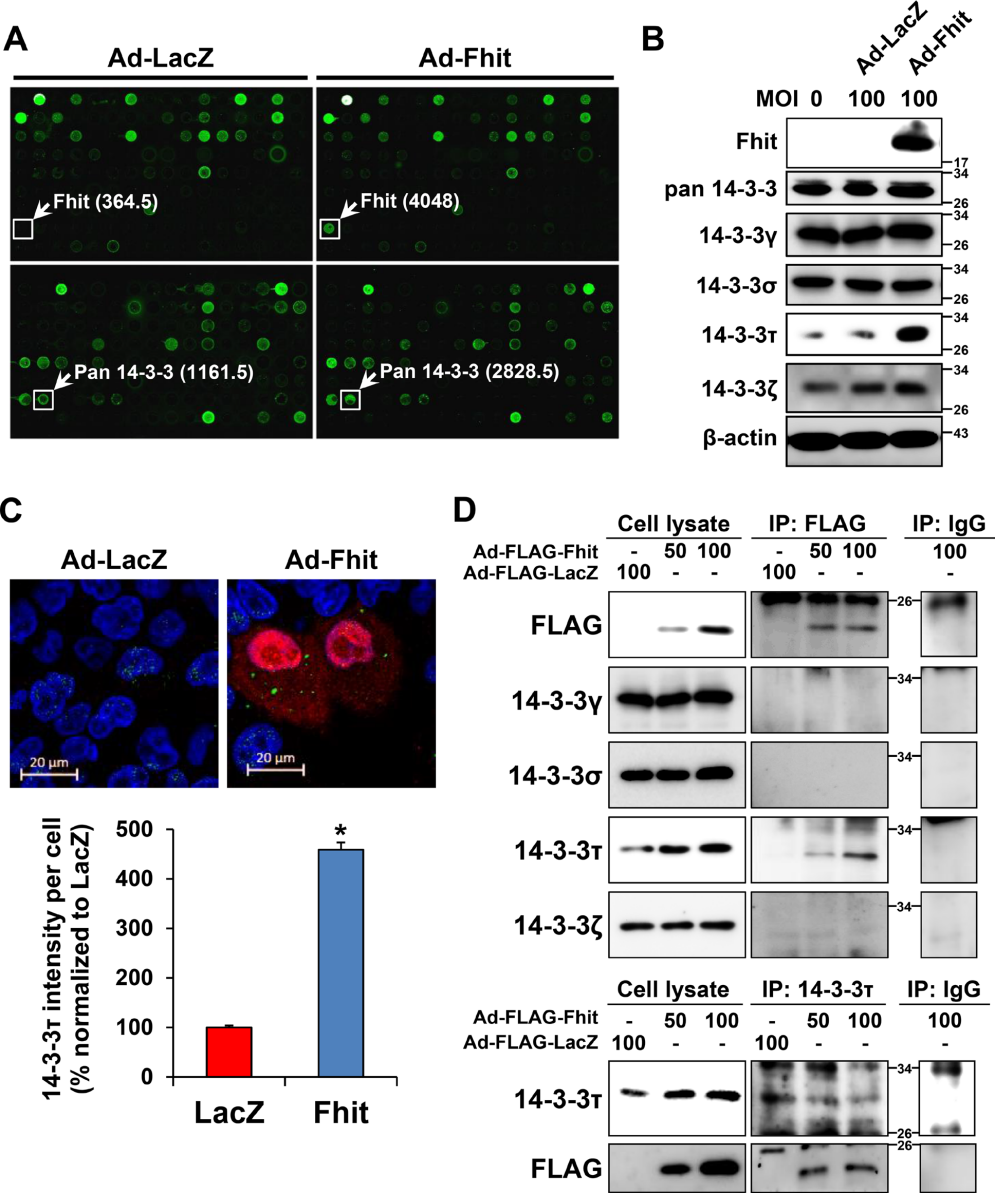
Up-regulation of a 14-3-3 protein in Ad-Fhit-transduced NSCLC cells (**A**) Antibody arrays of cell lysates from H460 cells infected with Ad-Fhit or Ad-LacZ. Numbers in parentheses represent signal intensity. (**B**) Expression of 14-3-3 isoforms was assessed by Western blot analysis in H460 cells infected with Ad-Fhit or Ad-LacZ. MOI, multiplicity of infection. (**C**) Immunofluorescence images of Fhit and 14-3-3τ expression in H460 cells infected with Ad-Fhit or Ad-LacZ (upper panel). Nuclei were stained with Hoechst 33342 (blue). Expression of Fhit protein is shown in red, and expression of 14-3-3τ protein is shown in green. The intensity of 14-3-3τ staining per cell was assessed (lower panel). Results are presented as mean percentage ± standard deviation (*n* = 3). *p < 0.05. (**D**) Immunoprecipitation analysis for the interaction of Fhit and 14-3-3τ proteins in NSCLC cells. Cell extracts were prepared from H460 cells transduced with Ad-Fhit, and immunoprecipitations (IP) were carried out by incubating the cell lysates with anti-FLAG antibody followed by immunoblotting with 14-3-3 antibodies, and vice versa.

A high-throughput ELISA-based antibody microarray was performed to identify candidate proteins that may be involved in Fhit-induced autophagy. Expression of proteins related to intracellular signal transduction, cytoskeletal regulation, mesenchymal transition, and apoptotic regulation was significantly altered within the 656 proteins analyzed in H460 cells transduced with Ad-Fhit (Table 1). Among these differently expressed proteins, the expression of 14-3-3 proteins was increased 1.8-fold compared with the LacZ control (Figure 3A). The 14-3-3 proteins have seven mammalian isoforms (β, γ, ε, η, σ, τ, ζ) and these isoforms are able to bind numerous protein ligands that play important roles in various cellular signal regulatory processes [22–24]. Especially, τ and ζ isoforms were reported to play a regulatory role in autophagy signaling [25, 26]. Therefore, we analyzed the expression of 14-3-3 isoforms after Ad-Fhit transduction. The level of 14-3-3τ protein was markedly increased, but those of γ, σ, and ζ isoforms were not changed significantly by Fhit overexpression (Figure 3B). The same results were observed in other NSCLC cell lines (Supplementary Figure 2A). We also performed immunofluorescence image analysis of Fhit and 14-3-3τ protein expression in H460 cells transduced with Ad-Fhit. 14-3-3τ protein expression was detected in the Fhit-expressing cells (Figure 3C). Additionally, we examined the effect of serum starvation on 14-3-3τ expression in Fhit-expressing or Fhit-deficient NSCLC cells. An increase in 14-3-3τ expression after serum starvation was detected only in Fhit-expressing cells (Supplementary Figure 2B). Further, we investigated the interactions between Fhit and 14-3-3τ in Ad-Fhit-transduced H460 cells using immunoprecipitation (Figure 3D). The endogenous 14-3-3τ protein was detected in FLAG-immunoprecipitated complexes (upper panel) and FLAG-tagged Fhit proten was also detected in immunoprecipitates pulled down by the anti-14-3-3τ antibody (lower panel), indicating an interaction between Fhit and 14-3-3τ proteins.

**Table 1:** Top 20 increased or decreased proteins upon transduction of the *Fhit* gene

Names of proteins	Fold changes	Names of proteins	Fold changes
Increased		Decreased	
Fhit	8.484	APC	0.458
Tubulin-a	2.516	Collagen II	0.495
Filamin	2.487	IL-6	0.586
Keratin 5/8	2.403	Cdk1/p34cdc2	0.599
Tubulin	1.871	bcl-X	0.604
Prohibitin	1.866	hNIS	0.653
CD16	1.850	bcl-2a	0.685
14.3.3, Pan	1.843	Erk1	0.712
Adrenocorticotrophic Hormone	1.813	DR5	0.730
Vimentin	1.802	Cdk8	0.739
Mast Cell Chymase	1.798	CA125	0.747
Keratin, Pan	1.790	Nck	0.748
Keratin, Multi	1.735	CA19-9	0.756
Melanoma (gp100)	1.711	JKK1	0.761
CD63	1.691	IgG	0.761
XPG	1.617	GST	0.772
Laminin B1/b1	1.608	GAPDH	0.773
Retinol Binding Protein	1.603	BRCA2 (aa 1323-1346)	0.785
Pds1	1.590	GSK-3	0.786
Notch	1.569	Bcl10/CIPER/CLAP/mE10	0.786

### 14-3-3τ is required for Fhit-induced autophagy in NSCLC cells

**Figure 4:**
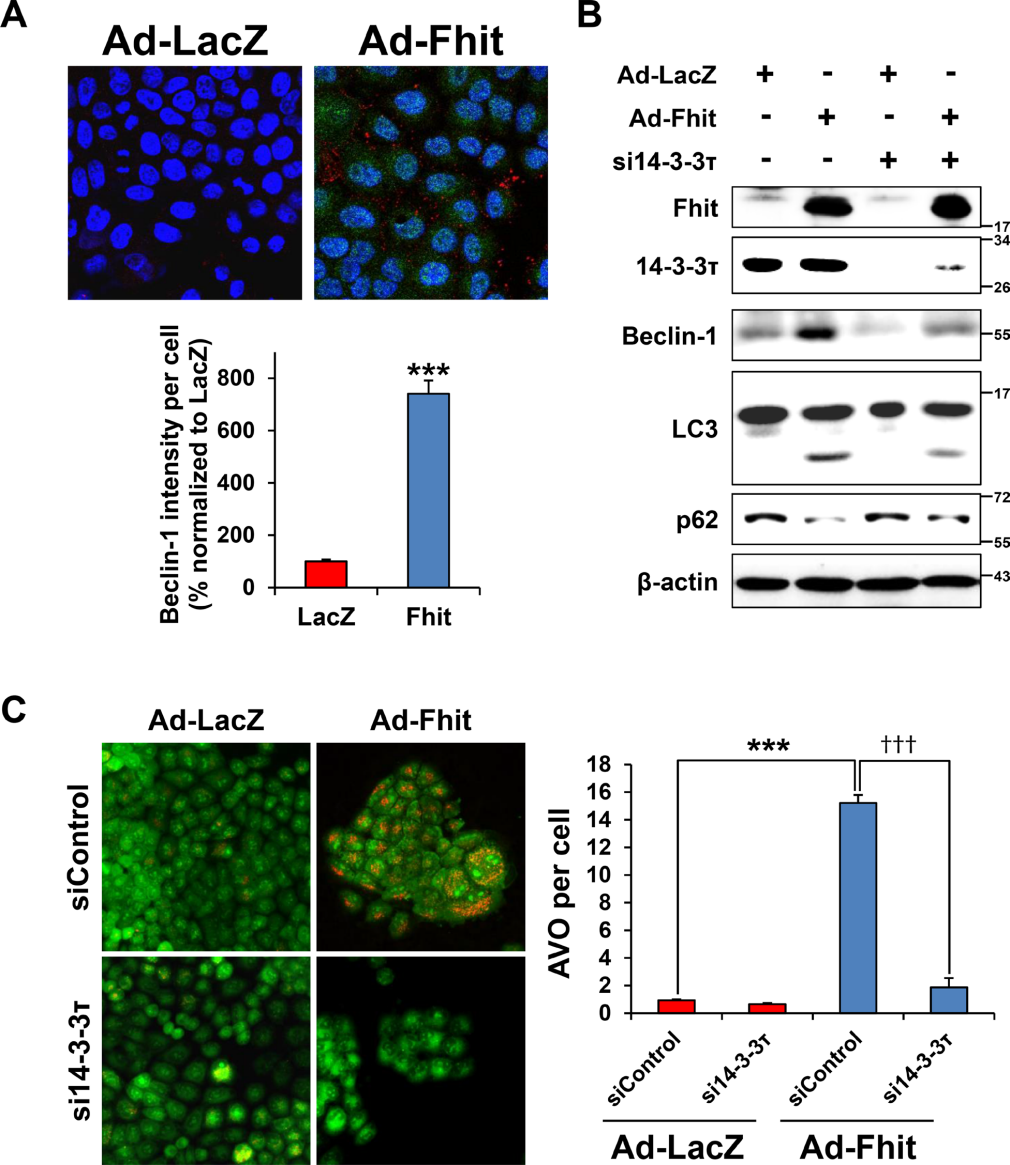
14-3-3τ is required for Fhit-induced autophagy (**A**) Immunofluorescence images of Fhit and beclin-1 expression in H460 cells infected with Ad-Fhit or Ad-LacZ (upper panel). Nuclei were stained with Hoechst 33342 (blue). Expression of Fhit protein is shown in green, and expression of beclin-1 protein is shown in red. The intensity of beclin-1 staining per cell was assessed (lower panel). Results are presented as mean percentage ± standard deviation (*n* = 5). ***p < 0.001. (**B**) Effect of 14-3-3τ silencing on expression of autophagy marker proteins. H460 cells were pretreated with 14-3-3τ siRNA (si14-3-3τ) for 24 h and then transduced with Ad-Fhit or Ad-LacZ for 48 h. Autophagy marker proteins were assessed by Western blot analysis. Scrambled siRNA was used as a nonspecific siRNA control. (**C**) Acridine orange staining in Ad-Fhit- or Ad-LacZ-infected H460 cells with or without pre-treatment with siRNA against 14-3-3τ (si14-3-3τ). Acidic vesicular organelles (AVO) were analyzed by confocal fluorescent microscopy (×200) (left panel). The number of AVO per cell was assessed (right panel). Data are presented as mean ± standard deviation of six independent experiments. ***p < 0.001 and ^†††^*p* < 0.001. siControl, contol siRNA.

A previous study showed that 14-3-3τ promotes transactivation of the beclin-1 promoter [25], suggesting a role of 14-3-3τ in Fhit-induced autophagy. Immunofluorescence analysis showed increased expression of beclin-1 in Fhit-transduced cells (Figure 4A). Next, to investigate whether the 14-3-3τ up-regulation observed after Fhit transduction is critical for Fhit-induced autophagy, we examined the effect of siRNA against 14-3-3τ on levels of autophagy markers in Ad-Fhit-infected H460 cells. Expression of beclin-1 and LC3-II and degradation of p62 were significantly reduced by inhibiting 14-3-3τ expression (Figure 4B). Formation of AVO was also remarkably decreased in Ad-Fhit-infected cells by treatment with 14-3-3τ siRNA (Figure 4C). These results indicate that 14-3-3τ up-regulation is critical in Fhit-induced autophagy.

### Inhibiting autophagy enhances Fhit-induced cell death in Fhit-deficient NSCLC cells

**Figure 5:**
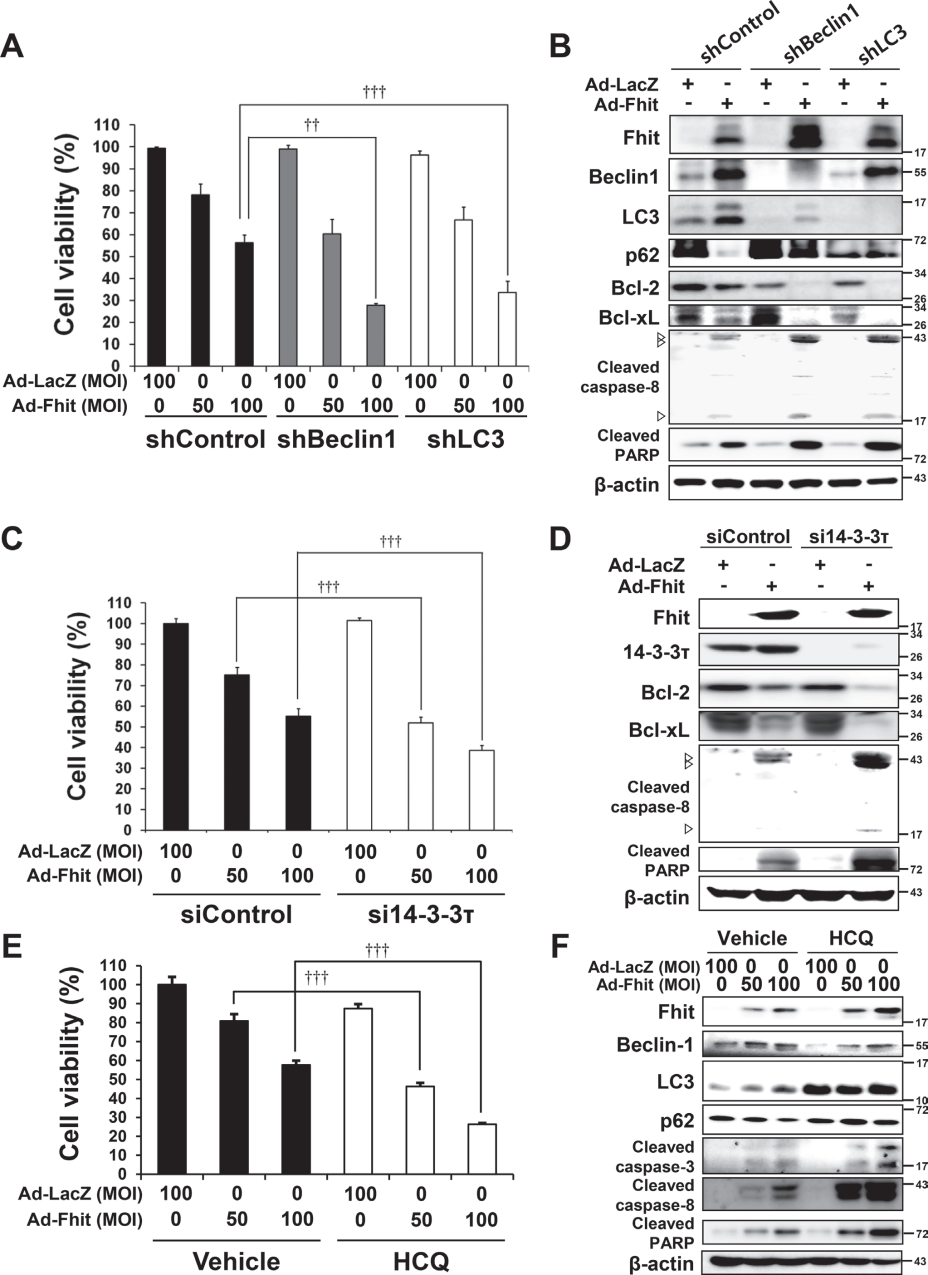
Inhibition of autophagy enhances Fhit-induced cell death (**A**) Effects of autophagy inhibition on viability of H460 cells transduced with Ad-Fhit. H460 cells, stably expressing shRNA against beclin-1 (shBeclin-1) or LC3B (shLC3) to block autophagy, were transduced with Ad-Fhit and cell viability was analyzed by a WST assay. Results are mean percentage ± standard deviation of three independent experiments. ^††^*p* < 0.01 and ^†††^*p* < 0.001 for the Fhit transduction with beclin-1 or LC3 shRNA treatment versus the Fhit transduction with control shRNA (shControl) treatment. MOI, multiplicity of infection. (**B**) Western blot analysis of apoptotic protein expression after Fhit transduction in H460 cells stably expressing shRNA against beclin-1 (shBeclin-1) or LC3B (shLC3). shControl, control shRNA. (**C**) Effects of 14-3-3τ inhibition on viability of H460 cells transduced with Ad-Fhit. H460 cells were transfected with siRNA against 14-3-3τ (si14-3-3τ) and then transduced with Ad-Fhit. Cell viability was analyzed by a WST assay. Scrambled siRNA (siControl) was used as a nonspecific siRNA control. Results are mean percentage ± standard deviation of three independent experiments. ^†††^*p* < 0.001 for the Fhit transduction with 14-3-3τ siRNA treatment versus the Fhit transduction with control siRNA treatment. MOI, multiplicity of infection. (**D**) Western blot analysis of apoptotic protein expression after *Fhit* transduction in H460 cells that had been transfected with siRNA against 14-3-3τ (si14-3-3τ). siControl, control siRNA. (**E**) Effects of pharmacological inhibition of autophagy by hydroxychloroquine (HCQ) on the viability of H460 cells transduced with Ad-Fhit. H460 cells were transduced with Ad-Fhit and then treated with 10 μM of HCQ or PBS (vehicle) as a control. Cell viability was assessed by a WST assay. Results are presented as mean percentage ± standard deviation of three independent experiments. ^†††^*p* < 0.001 for the Fhit transduction with HCQ treatment versus the Fhit transduction with vehicle treatment. (**F**) Western blot analysis of apoptotic protein expression in H460 cells that were transduced with Ad-Fhit and treated with 10 μM of HCQ.

To examine whether Fhit-induced autophagy functions to promote cell survival or cell death, we established stable autophagy-depleted H460 cells by lentiviral transduction of either beclin-1- or LC3B-specific shRNA. Cell viability of autophagy-depleted cells after Ad-Fhit infection was significantly decreased compared to control cells (Figure 5A). We also performed Western blot analysis to examine apoptosis marker proteins and found that Fhit-induced apoptosis was remarkably enhanced in autophagy-depleted H460 cells (Figure 5B). Next, we examined the effect of 14-3-3τ siRNA on cell viability of Ad-Fhit-infected H460 cells. Cell viability was significantly decreased in 14-3-3τ-inhibited cells compared with control cells after Ad-Fhit transduction (Figure 5C). Increase in Fhit-induced apoptosis was detected in cells transfected with siRNA against 14-3-3τ (Figure 5D). We also showed that treatment with the autophagy inhibitor hydroxychloroquine (HCQ) significantly decreased the cell viability of Ad-Fhit-infected H460 cells (Figure 5E). Moreover, an increase in Fhit-induced apoptosis was detected in cells treated with HCQ (Figure 5F). Taken together, these results indicate that Fhit-induced autophagy plays a cytoprotective role in Fhit-deficient NSCLC cells.

### Tumor growth is suppressed by intratumoral *Fhit* injection, and this antitumor effect is enhanced by inhibiting autophagy in a lung cancer xenograft model

**Figure 6:**
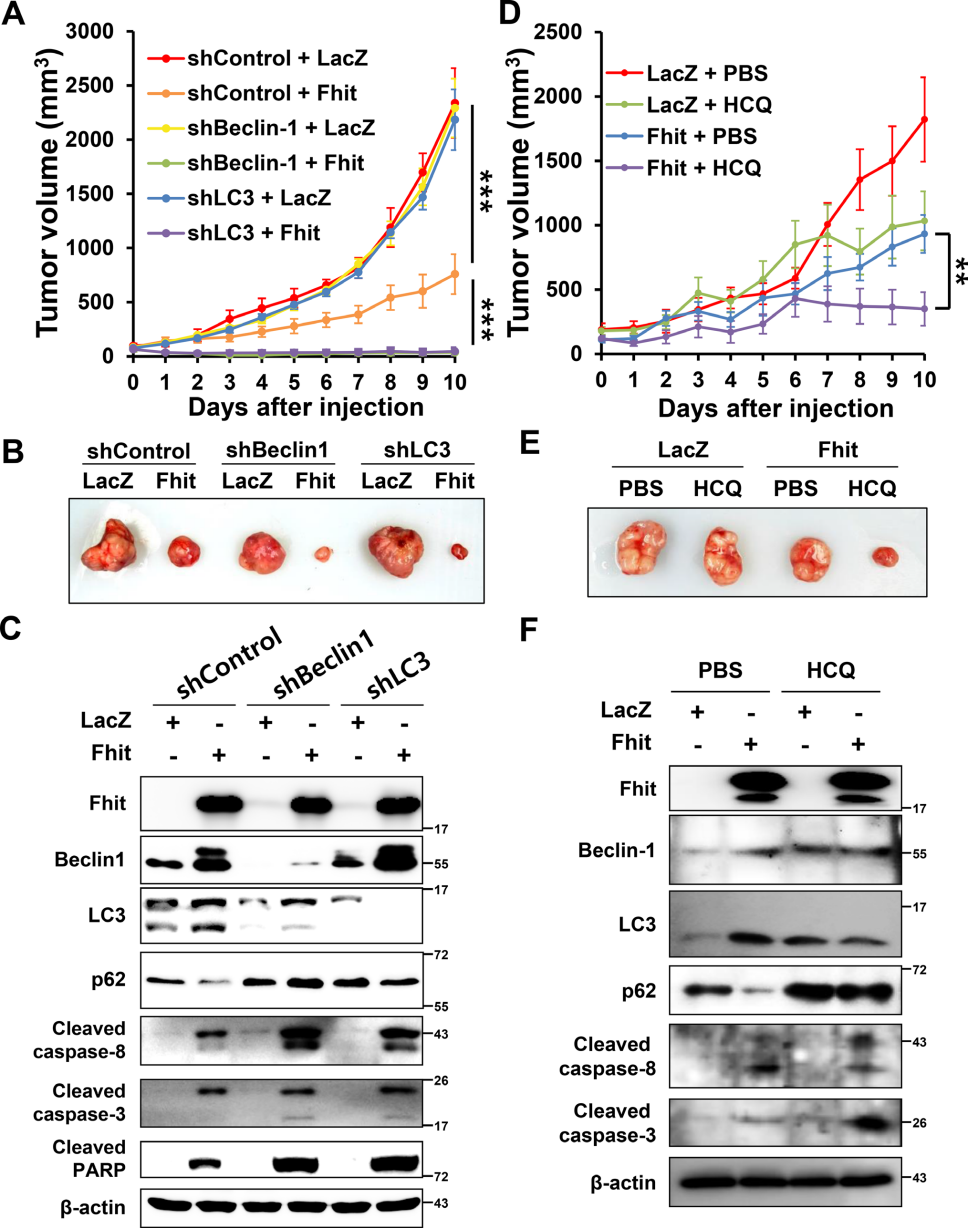
Inhibition of autophagy enhances the antitumor effect of Fhit protein *in vivo*. (**A**) Effects on tumor growth. H460 cells, stably expressing shRNA against beclin-1 (shBeclin-1) or LC3B (shLC3), were xenografted in nude mice. After tumors reached a volume of 100 mm^3^, a lentiviral vector expressing the *Fhit* gene (Fhit) or *LacZ* (LacZ) was injected into the tumors. Tumor volumes were measured daily. Results are reported as the mean ± standard deviation from six mice in each group. ***p < 0.001 for the control shRNA (shControl) + LacZ group versus the shControl + Fhit group and for the shControl + Fhit group versus the shBeclin-1 or shLC3 + Fhit group. (**B**) Tumors formed in nude mice. (**C**) Expression of apoptotic and autophagic proteins in tumors. Crude protein lysates were prepared from tumor samples on day 11 after injection and analyzed by Western blotting. (**D–F**) Effects of pharmacological inhibition of autophagy on the antitumor effect of Fhit protein. Hydroxychloroquine (HCQ, 60 mg/kg of body weight) or PBS was injected into the peritoneal cavity daily after intratumoral injection of lentiviral-Fhit (Fhit) or lentiviral-LacZ (LacZ). Results are shown as the mean ± standard deviation from five mice in each group. **p < 0.01 for the Fhit + PBS group versus Fhit + HCQ group.

To validate the observations from our *in vitro* experiments, we carried out *in vivo* experiments using a H460 tumor xenograft mouse model. Lentiviral-*Fhit* infection clearly resulted in a two-fold reduction in tumor volume compared with lentiviral-*LacZ*, and this antitumor effect was markedly increased in xenografts of autophagy-suppressed H460 cells which stably express shRNA against beclin-1 or LC3B (Figure 6A, 6B). Western blot analysis revealed significant increases in the expression of apoptotic markers in tumor samples derived from the autophagy-suppressed cells (Figure 6C). We also determined the effect of HCQ, an autophagy inhibitor, on the antitumor activity of Fhit *in vivo*. Mice treated with both HCQ and lentiviral-*Fhit* showed significantly reduced tumor growth compared with mice that did not receive HCQ treatment (Figure 6D, 6E). Western blot analysis of tumor samples derived from HCQ-treated mice revealed a significant increase in the expression of apoptotic markers (Figure 6F).

## DISCUSSION

The process of autophagy is initiated by the formation and expansion of membranous structures called phagophores. Phagophores gradually grow into double-membrane vesicles called autophagosomes that sequester cellular waste. Stimulation of the beclin-1 complex, a regulatory platform, generates phosphatidylinositol-3-phosphate, which promotes autophagosomal membrane nucleation. Autophagosomal elongation requires two ubiquitin-like conjugation systems: the Atg5–Atg12 conjugation system and the microtubule-associated protein light chain 3 (LC3–Atg8) conjugation system. LC3 is a protein located on the inner membrane of autophagosome. The conversion of a cytosolic truncated form of LC3 (LC3-I) to its autophagosomal membrane-associated, phosphatidylethanolamine-conjugated form (LC3-II) is correlated with the progression of autophagosome formation. Therefore, LC3 is one of the most important markers of autophagy. In addition, p62 is the ligand of LC3, and its degradation is also considered to be a marker for autophagy. After the autophagosome fuses to the lysosome, the cargo with the inner membrane of the autophagosome enters the lysosome and is degraded by lysosomal enzymes [12–15].

The 14-3-3 proteins constitute a highly conserved family of homodimeric and heterodimeric molecules that are expressed in all eukaryotic cells. They bind serine/threonine-phosphorylated intracellular proteins and participate in important cellular events including regulation of apoptosis, cell cycle control, DNA repair, mitogenic signal transduction, and transcriptional regulation. In human cells, this family of proteins consists of seven isoforms: β, γ, ε, η, σ, τ, and ζ [22–24].

Several candidate Fhit-interacting proteins have been reported, including Mdm2, α-tubulin, and b-catenin [27–29]. However, a direct interaction between Fhit and these proteins has not yet been detected by co-immunoprecipitation. In this study, we found by an antibody microarray that Fhit overexpression induces up-regulation of 14-3-3 proteins, and confirmed that the 14-3-3τ isoform is the specific isoform increased that plays a crucial role for Fhit-induced autophagy in NSCLC cells. Further, we showed interaction between Fhit and 14-3-3τ using immunoprecipitation (Figure 3D). It was previously reported that 14-3-3 proteins play a regulatory role in autophagy signaling by regulating target proteins of the autophagic machinery [30, 31]. It was also reported that beclin-1, a well-known key regulator of autophagy, contains a 14-3-3 protein binding motif, and that endogenous 14-3-3 and beclin-1 co-immunoprecipitate in HeLa cells [32]. Taken together, these data suggest that 14-3-3 proteins may regulate beclin-1 and thus regulate autophagy. With regard to specific 14-3-3 isoforms, there was a report showing that 14-3-3τ up-regulates beclin-1 expression, probably through E2F1, regulating autophagy in cancer cells [25]. This study showed that depletion of 14-3-3τ inhibits beclin-1 expression in many cell lines, whereas up-regulation of 14-3-3τ induces beclin-1 expression. The expression of beclin-1 depended on E2F1, and E2F1 could transactivate the beclin-1 promoter. Depletion of E2F1, like 14-3-3τ, also inhibited autophagy. This observation is concordant with our results; Fhit-induced autophagy is associated with up-regulation of both 14-3-3τ and beclin-1, and inhibition of 14-3-3τ causes a decrease in beclin-1 levels (Figure 4). Further studies are needed to identify downstream targets of the Fhit protein and the detailed mechanisms of signal transduction.

Meanwhile, it was previously reported that viral-mediated Fhit restoration increases the production of intracellular reactive oxygen species, followed by increased apoptosis of lung cancer cells under oxidative stress conditions [33]. Additionally, numerous studies have reported that reactive oxygen species act as early inducers of autophagy upon nutrient deprivation [34]. Therefore, reactive oxygen species may modulate Fhit-induced autophagy in lung cancer cells.

The net effect of autophagy on cell death is contextual, and both cytoprotective and cytotoxic functions of autophagy have been reported [16–18]. Autophagy comprises a pro-survival stress response; for example, during nutrient deprivation it serves to ensure energy balance. However, there is also evidence showing that over-activation of autophagy can lead to cell death. There are many reports of cross-talk between apoptosis and autophagy pathways [35–38], and autophagy can play either a pro-death or pro-survival role through interplays with apoptosis. Under certain conditions, autophagy activation inhibits apoptosis. In other situations, autophagy occurs upstream of apoptosis and activates it. This study showed that autophagy induced by *Fhit* transduction acts as a cell survival mechanism, because autophagy inhibition caused an increase in apoptotic cell death (Figure 5).

It was previously reported that the role of autophagy in pancreatic tumor development is connected to the status of the tumor suppressor p53 [39]. In the report, progression to pancreatic tumor was blocked in mice lacking the essential autophagy genes Atg5 or Atg7. However, in mice that contain oncogenic Kras and lack p53, loss of autophagy no longer blocked tumor progression, but accelerated tumor formation. We therefore analyzed whether the p53 status affects Fhit-induced autophagy. Our experiments showed that the cytoprotective role of Fhit-induced autophagy was not different in H358 cells, which are deficient in p53 expression, compared to that in H460 cells (Supplementary Figure 3).

The autophagy inhibitor HCQ is a candidate therapy for cancer treatment and is currently being tested in several clinical trials [40]. We therefore performed the experiment with a combination of HCQ treatment and Fhit overexpression, in addition to inhibiting autophagy via siRNAs. We showed that Fhit-induced cell death increased with HCQ treatment.

To the best of our knowledge, this study is the first to describe that overexpression of Fhit, a tumor suppressor protein, induces autophagy in NSCLC cells. Further, we found that this autophagy is mediated by 14-3-3τ and plays a cytoprotective role against the antitumor effect of Fhit both *in vitro* and *in vivo*. Therefore, suppressing autophagy might be a promising therapeutic option to enhance the efficacy of *Fhit* gene therapy in NSCLC, and further studies are needed.

## MATERIALS AND METHODS

### Antibodies

The anti-Fhit antibody was purchased from Invitrogen (Waltham, MA, USA). Antibodies against bcl-2, cleaved caspase-3, cleaved caspase-8, cleaved PARP, beclin-1, LC3B, and DYKDDDDK (FLAG) tag were purchased from Cell Signaling Technology (Beverly, MA, USA). Antibodies against bcl-xL, pan 14-3-3 (K-19), 14-3-3γ (KC21), 14-3-3σ (5D7), 14-3-3τ (C-17), and 14-3-3ζ (C-16) were purchased from Santa Cruz Biotechnology (Santa Cruz, CA, USA). The anti-p62 antibody was obtained from Abnova (Taipei, Taiwan). Anti-β-actin and anti-FLAG M2 antibodies were purchased from Sigma (St. Louis, MO, USA).

### Cell culture

Calu-3, HCC827, NCI-H358, NCI-H460, and NCI-H1299 NSCLC cell lines were obtained from the American Type Culture Collection (Manassas, VA, USA). Calu-3 and HCC827 cells express endogenous Fhit protein, but H358, H460, and H1299 cells are deficient for Fhit [10, 41, 42]. Calu-3 cells were cultured in DMEM; and HCC827, H358, H460, and H1299 cells were cultured in RPMI supplemented with 10% fetal bovine serum (FBS) and 100 μg/ml streptomycin at 37°C under 5% CO_2_. All cell culture materials were purchased from HyClone Laboratories (Logan, UT, USA). For serum starvation, cells were washed with serum-free media twice and then cultured in DMEM or RPMI with no FBS for the indicated time periods.

### Recombinant *Fhit* adenoviral vector and transduction

Human *Fhit* (GenBank accession number NM_001166243.1) cDNA was subcloned from pRc/CMV-Fhit into HindIII and XbaI-restricted p3xFLAG-CMV-7.1 vector (Sigma). The adenoviral *Fhit* (Ad-Fhit) vector was prepared using the pAdEasy System (Qbiogene; Carlsbad, CA, USA) according to the manufacturer's instructions and as described previously [43]. An adenoviral *LacZ* (Ad-LacZ) vector was used as a control (Qbiogene). For infection, NSCLC cells were treated with Ad-Fhit or Ad-LacZ at 100 multiplicity of infection (MOI) and incubated for 48 h.

### CRISPR/Cas9 knockout (KO) plasmid transfection for *in vitro* *Fhit* gene knockout

The CRISPR/Cas9 system can be used to generate knockout cells by co-expressing a guide RNA (gRNA) specific to the target gene and the endonuclease Cas9 [44]. Fhit CRISPR/Cas9 KO plasmid containing Cas9 nuclease and a pool of three *Fhit*-specific 20 nt gRNAs was purchased from Santa Cruz Biotechnology. gRNA sequences are derived from the GeCKO v2 libraries. HCC827 (Fhit-positive) NSCLC cells were transfected with the Fhit CRISPR/Cas9 KO plasmid and Fhit homology-directed repair (HDR) plasmid (Santa Cruz Biotechnology) containing a puromycin resistance gene for the selection of cells with DNA double strand breaks (DSB) in the *Fhit* gene. Transfection of the plasmids was carried out using UltraCruz^®^ Transfection Reagent (Santa Cruz Biotechnology) according to the manufacturer's instructions. Media were replaced with fresh media containing 2 μg/ml puromycin 48 h post-transfection for stable selection of *Fhit*-knockout cells.

### Lentiviral short hairpin RNA (shRNA) production and cell transduction

pLKO.1 lentiviral vectors expressing shRNA against beclin-1 (RHS4533-EG8678) or LC3B (RHS4533-EG81631) were purchased from GE Dharmacon (Lafayette, CO, USA). Lentiviral vectors were transfected into HEK 293T cells cultured in DMEM supplemented with 10% FBS, and supernatants containing viral particles were collected after 48 h. Then, the virus were filtered through 0.45-μm membranes (Millipore; Darmstadt, Germany) and stored immediately at -70°C. Viral titer was determined by the TCID_50_ method. H460 cells were transduced with 20 MOI of virus. One day post-infection, media were removed and replaced with media containing puromycin (2 μg/ml) for selection of stable lines.

### Small interfering RNA (siRNA) transfection

A pool of three 14-3-3τ-specific 19-25 nt siRNAs (sc-29586) and non-targeting control siRNA were purchased from Santa Cruz Biotechnology. Transfection of siRNAs was carried out using Oligofectamine™ (Invitrogen; Carlsbad, CA, USA) according to the manufacturer's specifications. Specific silencing of 14-3-3τ was confirmed by at least three independent experiments.

### Western blotting

Cell lysates were resolved by 10% SDS-PAGE and transferred to Immobilon-P polyvinylidene difluoride membranes (Millipore). The membranes were blocked with 5% skim milk–PBS–0.1% Tween 20 for 1 h at room temperature before being incubated overnight with primary antibodies diluted in 5% skim milk–PBS–0.1% Tween 20. The membranes were then washed three times in PBS–0.1% Tween 20 and incubated with horseradish peroxidase-conjugated secondary antibodies (Santa Cruz Biotechnology) diluted 1:5,000 in 5% skim milk–PBS–0.1% Tween 20 for 1 h. After successive washes, the membranes were developed using an ECL kit (Thermo Scientific; Waltham, MA, USA) and analyzed by the ImageQuant LAS 4000 image analysis system (GE Healthcare; Buckinghamshire, UK).

### Immunofluorescence microscopy

Ad-Fhit-infected cells were seeded on coverslips, grown overnight, and fixed with 4% paraformaldehyde in PBS for 15 min. Following permeabilization with 0.5% Triton X-100 in PBS for 10 min at room temperature, cells were then blocked with 3% BSA in PBS for 30 min at room temperature and incubated overnight at 4°C with primary antibodies. After three 5-min washes in PBS, cells were incubated for 1 h at room temperature with Alexa Fluor 488- or Alexa Fluor 568-conjugated secondary antibodies (Invitrogen). Then, cells were counterstained with Hoechst 33342 for 5 min for nuclear staining, washed twice in PBS, and mounted on glass slides using Vectashield (Vector Laboratories; Burlingame, CA, USA). Cells were visualized using a Zeiss LSM 700 laser scanning microscope (Carl Zeiss; Oberkochen, Germany). Quantification of proteins in the acquired images was performed by using ImageJ software (NIH Image; Bethesda, MD, USA).

### Quantification of acidic vesicular organelles (AVO) with acridine orange (AO) staining

Ad-Fhit-infected cells were seeded on coverslips. After 48 h of incubation, AO staining was performed. Briefly, the treated cells were stained with AO (1 μg/ml) in serum-free media in the dark at 37°C for 15 min and then were washed with serum-free media. AO staining was visualized immediately using a confocal microscope. To quantify AVO in cells, fluorescence-activated cell sorting (FACS) analysis was performed on AO-stained cells using a FACSCanto™ II flow cytometer (BD Biosciences; San Jose, CA, USA).

### Antibody array

Explorer Antibody Microarray (Full Moon Biosystems; Sunnyvale, CA, USA) was used according to the protocol suggested by the manufacturer. Briefly, 50 μg of purified Ad-Fhit- or Ad-LacZ-infected H460 cell lysate was incubated with 3 μl of a 10 μg/μl biotin/DMF solution for 90 min at room temperature. The array slide was treated with 30 ml of blocking solution for 1 h and then incubated with the biotin-labeled protein in 6 ml of coupling buffer for 2 h. After coupling, the slide was washed and incubated for 20 min with Cy3-streptavidin (GE Healthcare) diluted in detection buffer. After detecting, the slide was washed with washing solution and rinsed with ultrapure water. The slides were completely dried and scanned using a GenePix 4000B scanner (Molecular Devices; Sunnyvale, CA, USA) within 24-48 h. The scanned images were gridded and quantified with GenePix Software (Molecular Devices). Quantitative data were analyzed using Genowiz 4.0™ (Ocimum Biosolutions, India).

### Immunoprecipitation

Each cell lysate (500 μg), precleared with protein A–agarose beads (Santa Cruz Biotechnology), was incubated with 3 μg of anti-FLAG or anti-14-3-3τ antibody at 4°C overnight, immobilized on protein A–agarose beads, washed five times in lysis buffer, eluted by boiling, and subjected to SDS-PAGE and immunoblotting.

### Analysis of cell viability

H460 cells stably expressing shRNA against beclin-1 or LC3 were plated in 96-well plates and cultured for 24 h. Then, cells were treated with Ad-Fhit or Ad-LacZ virus at 100 MOI. For pharmacological inhibition of autophagy, cells were plated in 96-well plates, incubated overnight, transduced with 100 MOI of Ad-Fhit or Ad-LacZ, and then treated with 10 μM of HCQ (US Pharmacopeia; Rockville, MD, USA). After 48 h, cell proliferation was determined by a WST assay. WST1 reagent (DoGen Bio; Seoul, Korea), a water-soluble tetrazolium salt, was added to cells according to the manufacturer's instructions, and cells were incubated at 37°C for 1 h. Then, the optical density of each well was read at 450 nm.

### *In vivo* tumorigenicity and intratumoral injection of *Fhit* lentiviral vector

All animal experiments were approved by the Institutional Animal Care and Use Committee. Seven-week-old female athymic nude mice from the Nara Bio animal center (NARA Biotech; Seoul, Korea) were housed under pathogen-free conditions in microisolator cages with laboratory chow and water ad libitum. NSCLC xenografts were established by subcutaneously injecting 5 × 10^6^ cells per mouse mixed 1:1 with Matrigel basement membrane matrix (BD Biosciences) into their right flanks. Once tumors reached a volume of ~100 mm^3^, mice received an intratumoral injection of recombinant lentiviral-*Fhit* or lentiviral-*LacZ* vector (8 × 10^7^ IU in 50 μl). Three days later, each virus was reinjected into the tumor mass. For pharmacological inhibition of autophagy, HCQ (60 mg/kg of body weight) or PBS was injected into the peritoneal cavity daily. Six mice per group were treated with infected cells. Tumor volume was determined from caliper measurements of tumor length (L) and width (W) according to the formula LW^2^/2.

### Statistical analysis

All experiments were repeated at least three times. Data are presented as mean ± standard deviation as a percentage of control. The Mann-Whitney *U*-test was used for comparisons between groups and a *p* value of < 0.05 was considered statistically significant.

## SUPPLEMENTARY MATERIALS FIGURES


