# Supplementary material for: Fhit, a tumor suppressor protein, induces autophagy via 14-3-3τ in non-small cell lung cancer cells
Source: Oncotarget. 2017 Mar 29;8(19):31923–37. doi: 10.18632/oncotarget.16652 (PMC5458259; doi:10.18632/oncotarget.16652)
Supplement: Supplementary file 1 [file oncotarget-08-31923-s001.pdf]

Fhit, a tumor suppressor protein, induces autophagy via 14-3-3τ in non-small cell lung cancer cells

Supplementary Materials

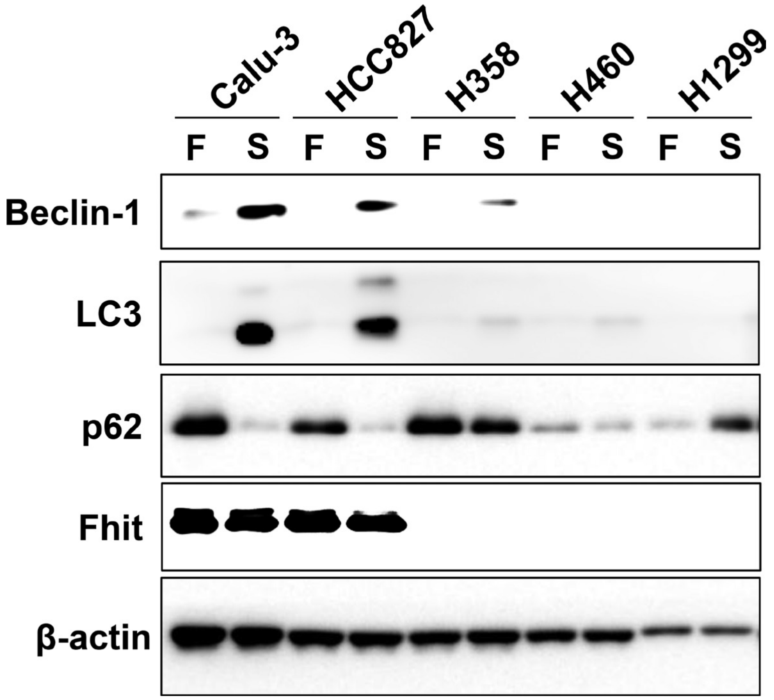

Supplementary Figure 1: Effect of serum starvation on autophagy in Fhit-expressing or Fhit-deficient NSCLC cells. Calu-3 and HCC827 cell lines harbor a wild-type *Fhit* gene and a deletion of the *TP53* gene. H358 and H1299 cell lines have deficient Fhit expression and a deletion of the *TP53* gene. The H460 cell line does not express Fhit and harbors a wild-type TP53 gene. Each cell line was cultured with or without serum. After 2 days, autophagy-related proteins were analyzed by Western blot analysis. F, culture with 10% FBS; S, serum-starved.

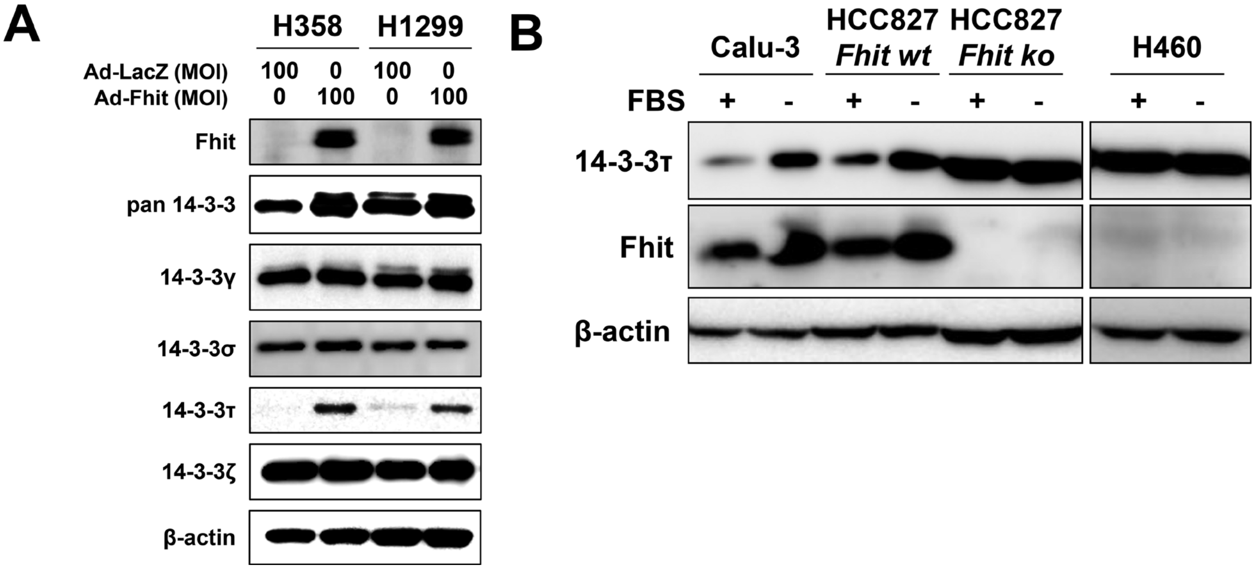

Supplementary Figure 2: Expression of *Fhit* gene up-regulates the τ isoform of 14-3-3 proteins in NSCLC cells. (**A**) Expression of 14-3-3 family members was analyzed by Western blot analysis in Ad-Fhit- or Ad-LacZ-transduced H358 and H1299 cells. (**B**) Effect of serum starvation on 14-3-3τ expression in Fhit-expressing or Fhit-deficient NSCLC cells. Calu-3 and HCC827 cells, harboring a wild-type *Fhit* gene, and H460 and *Fhit*-knockout HCC827 cells, that do not express Fhit, were cultured with or without serum. After 2 days, 14-3-3τ was analyzed by Western blot analysis. *wt*, wild type; *ko*, knockout; FBS, fetal bovine serum.

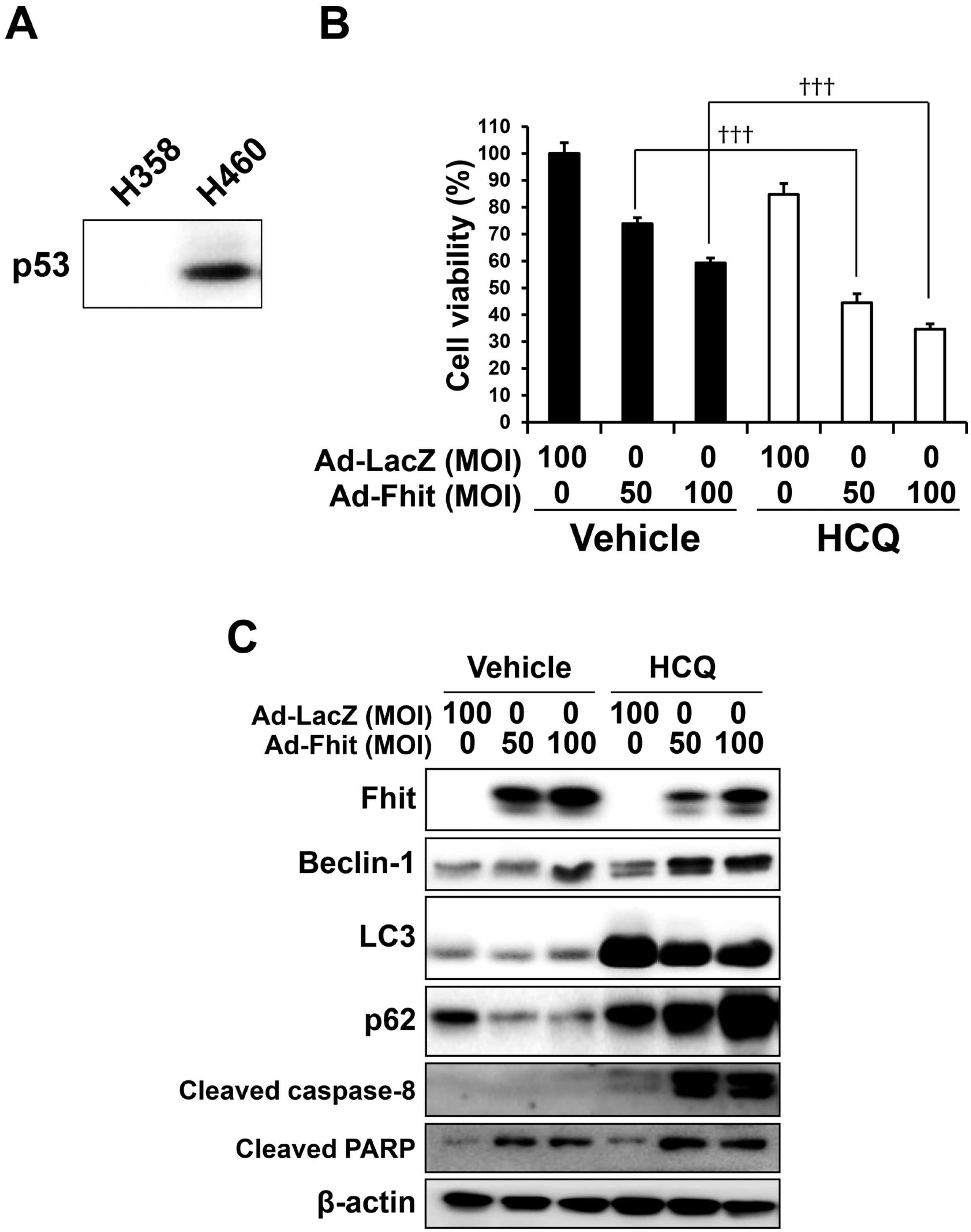

Supplementary Figure 3: Inhibition of autophagy enhances Fhit-induced cell death in Fhit- and p53-deficient H358 cells. (**A**) Western blot analysis of p53 protein in H358 and H460 cell lines. (**B**) Effects of pharmacological inhibition of autophagy by hydroxychloroquine (HCQ) on the viability of H358 cells. H358 cells were transduced with Ad-Fhit and then treated with 10 μM of HCQ or PBS (vehicle) as a control. Cell viability was assessed by a WST assay. Results are presented as mean percentage ± standard deviation of three independent experiments. $^{†††}p < 0.001$ for the Fhit transduction with HCQ treatment versus the Fhit transduction with vehicle treatment. (**C**) Western blot analysis of apoptotic protein expression in H358 cells that were transduced with Ad-Fhit and treated with 10 μM of HCQ.
